# The association the patient-reported outcomes after periacetabular osteotomy with radiographic features: a short-term retrospective study

**DOI:** 10.1186/s13018-021-02858-9

**Published:** 2021-12-19

**Authors:** Yinuo Fan, Weifeng Li, Yunlong Wu, Ruoyu Li, Guoju Hong, Zhongfeng Li, Lixin Chen, Hanjun Fang, Chi Zhou, Wei He, Zhenqiu Chen

**Affiliations:** 1 The First Clinical Medical College, Guangzhou University of Chinese Medicine, 12 Jichang Road, Baiyun District, Guangzhou, 510405 Guangdong Province People’s Republic of China; 2 The Department of Orthopedics, The First Affiliated Hospital of Guangzhou University of Chinese Medicine, Guangzhou, 510405 Guangdong Province People’s Republic of China; 3 Department of Joint Diseases, The Third Affiliated Hospital of Guangzhou University of Chinese Medicine, NO. 261 Longxi Road, Liwan District, Guangzhou, Guangdong Province People’s Republic of China; 4 Candidate, Research Fellow, Division of Orthopaedic Surgery, The University of Alberta, Edmonton, Canada; 5 Institute of Orthopedics, Guangzhou University of Chinese Medicine, Guangzhou, People’s Republic of China

**Keywords:** Bernese periacetabular osteotomy, Patient-reported outcomes, Multivariate logistic regression analysis, Receiver operating characteristic curve

## Abstract

**Background:**

Bernese periacetabular osteotomy (PAO) is an effective treatment for patients with developmental dysplasia of the hip (DDH). PAO has been widely used in China, but few follow-up outcomes have been reported in the international community. Moreover, the risk factors affecting patient-reported outcomes have not been discussed in recent studies. In this study, patient-reported outcomes after PAO were reported, and risk factors affecting patient-reported outcomes were analyzed.

**Methods:**

Patients who underwent PAO for DDH from January 2014 to January 2020 were selected as the study subjects, and 66 hips were included in the analysis after screening (59 patients, with an average follow-up time of 3.01 years). The Harris Hip Score (HHS) and International Hip Outcome Instrument-12 (iHOT-12) were used to assess hip function and patient quality of life. The changes of preoperative and latest follow-up HHSs less than 9 were defined as symptomatic hips, that is, an adverse outcome; otherwise, the score indicates preserved hips. Also, the changes of preoperative and latest follow-up iHOT-12 were defined as symptomatic hips and preserved hips. Multivariate logistic regression analysis was used to predict the risk factors influencing the patient-reported outcomes, and receiver operating characteristic (ROC) curve analysis was performed on the risk factors to determine their sensitivity, specificity and cutoff value.

**Results:**

Clinical outcome analysis demonstrates marked improvements in patient-reported outcomes. The multivariate logistic regression analysis showed that when the postoperative LCEA was > 38°, adverse outcomes were much more likely. However, a Tönnis angle of − 10° to 0° was a protective factor. In addition, hips with fair or poor joint congruency were more likely to develop negative outcomes. The ROC curve analysis showed that the optimal thresholds for the LCEA and Tönnis angles used to predict outcomes after PAO were 38.2° and − 9°, respectively. Based on the results of the ROC curve analysis, among hips with poor or fair joint congruency preoperatively treated by surgeons who obtained the improper postoperative LCEAs and Tönnis angles, bad patient-reported outcomes will most likely be obtained.

**Conclusions:**

Our results demonstrate marked improvements in patient-reported outcomes. Among hips with preoperative excellent or good joint congruency treated by experienced surgeons who obtain the proper postoperative LCEA and Tönnis angles, good patient-reported outcomes can be expected.

## Introduction

Developmental dysplasia of the hip (DDH) refers to inadequate coverage of the femoral head by the acetabulum due to abnormal structural development of the hip, which leads to subluxation or complete dislocation of the hip [1, 2]. Periacetabular osteotomy (PAO), first used by Ganzs, is one of the most commonly used hip-conserving surgeries for DDH [3]. PAO not only corrects the lateral, anterior coverage, forward and backward tilt, and inward and outward displacement of the acetabulum but also ensures continuity of the posterior column of the pelvis and provides satisfactory correction of the acetabulum in DDH patients, thereby delaying hip osteoarthritis and total hip arthroplasty (THA), rendering it fully applicable to young patients [4].

This surgery has been widely used in many countries, and some follow-up results have confirmed the efficacy of PAO [5–17]. China was one of the countries to adopt PAO early, but there are few follow-up studies on PAO, making current PAO studies important. In addition, we found that short-term follow-ups after PAO mostly addressed patient-reported outcomes with no discussion of risk factors affecting patient-reported outcomes [6, 8–11, 14, 15]. In contrast with these short-term follow-up results, our study not only performed an analysis of the impact of postoperative radiographic parameters on patient-reported outcomes but also performed ROC analysis on the postoperative radiographic parameters to determine their cutoffs.

Considering the current situation, this study retrospectively analyzed the data of DDH patients treated with PAO from 2014 to January 2020. The purpose of this study was to evaluate the short-term efficacy of PAO for DDH, analyze the association the patient-reported outcomes after PAO with radiographic features, and propose measures to improve patient-reported outcomes.

## Materials and methods

### General information

**Fig. 1:**
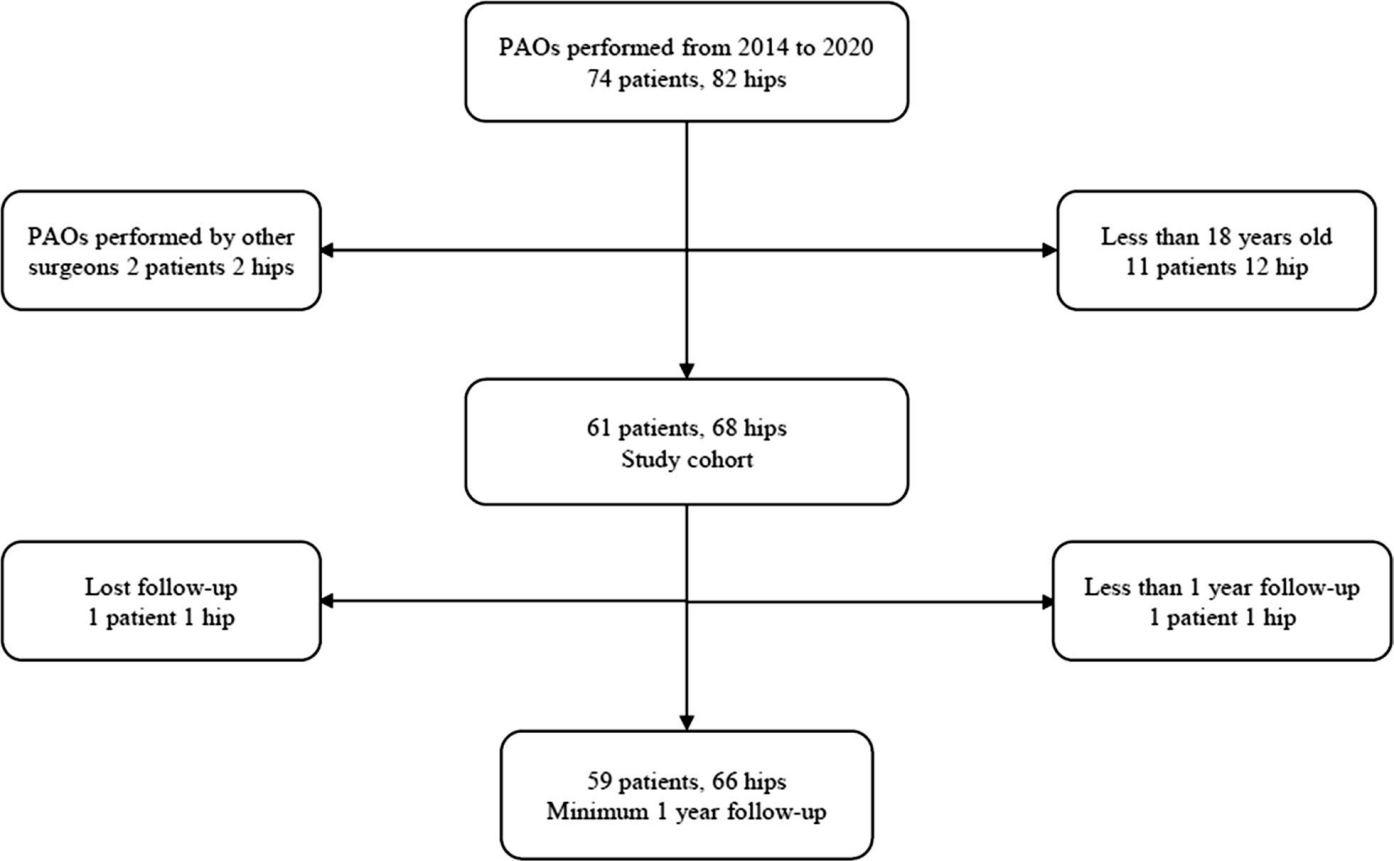
Flowchart of screening cases

After approval by the Institutional Review Committee, patients who underwent PAO for DDH in the First Affiliated Hospital of Guangzhou University of Chinese Medicine from 2014 to January 2020 were selected as study subjects. The diagnosis of DDH was determined by highly qualified physicians through radiographic evidence and symptoms. The inclusion criteria were: (1) radiographic evaluation was lower than Tönnis grade 3; (2) younger than 55 years old and older than 18 years old; (3) complete follow-up data and follow-up time is at least 1 year; (4) all DDH patients who met these criteria underwent PAO. The exclusion criteria were as follows: (1) isolated acetabular retroversion, neuromuscular or connective-tissue disorder, Legg–Calve–Perthes disease, or DDH combined with other diseases, such as Legg–Calve–Perthes disease or acetabular retroversion; (2) Tönnis grade 3 or other hip trauma; and (3) patients with no follow-up due to interrupted communication. The screening process of cases is shown in Fig. 1.

### Preoperative and postoperative examination

Before PAO, the functional activity of the hip and quality of life of the patient were evaluated by Harris Hip Score (HHS) [18] and International Hip Outcome Tool-12 (iHOT-12) [19], and a standard anteroposterior radiograph of the pelvis was routinely taken. After PAO, the HHS and iHOT-12 ratings were evaluated, and a standard anteroposterior radiograph of the pelvis was taken.

### Outcome measures

The HHS and iHOT-12 were used to evaluate the joint activity, pain, and quality of life in preoperative and postoperative patients with PAO. HHSs can be divided into four grades: excellent (≥ 90), good (80–89), fair (70–79), and poor (< 70) and include measures of pain, function, joint movement, limb deformity, etc. In this study, adverse outcomes were defined through HHSs. According to previous study [20], it has been found that the minimal clinically important difference (MCID) is 7–9 for the HHS and 13 for the iHOT-12 [21]. Thus, the changes of preoperative and latest follow-up HHSs less than 9 were defined as symptomatic hips, that is, an adverse outcome; otherwise, the score indicates preserved hips. Also, the changes of preoperative and latest follow-up iHOT-12 were defined as symptomatic hips and preserved hips. From August to September 2020, all included patients were administered a questionnaire.

The lateral center-to-edge angle (LCEA), acetabular coverage ratio, sharp angle and Tönnis angle were used to evaluate the radiographic correction degree during the PAO. LCEA, acetabular coverage ratio, sharp angle and Tönnis angle were measured by Digimizer software (version 5.4.6) before and after the PAO. DDH is generally believed to exist with an LCEA < 20° [22, 23], acetabular coverage ratio < 75% [24], sharp angle > 40° [25] and Tönnis angle > 10° [26]. In addition, according to previous studies [22, 23], the preoperative LCEA was graded as follows: LCEA < 5°, 5° < LCEA < 20°, and LCEA > 20°. According to Wells et al. [17], a postoperative LCEA was graded as 20° < LCEA < 38° and LCEA > 38°. There is no uniform standard for the range of correction for the Tönnis angle after PAO. According to the experience of the surgeons involved with this study, the postoperative Tönnis angle was within the appropriate range at 0 ± 10°. Therefore, we graded the postoperative Tönnis angle as 0 < Tönnis angle < 10°, − 10° < Tönnis angle < 0 and Tönnis angle < − 10°. All of these indicators were measured and observed by three authors, and the final results were averaged.

The Tönnis classification of hip osteoarthritis [27, 28] was divided into four grades: 0 (no signs of osteoarthritis), 1 (slight narrowing of the joint space, slight lipping at the joint margin, and slight sclerosis of the femoral head or acetabulum), 2 (small cysts in the femoral head or acetabulum, moderate narrowing of the joint space, and moderate loss of sphericity of the femoral head), and 3 (large cysts, severe narrowing or obliteration of the joint space, severe deformity of the femoral head, and avascular necrosis). Joint congruency [29] was described by excellent (radii of curvature of the acetabulum and femoral head identical and joint space maintained), good (curvature of the femoral head and acetabulum not identical, but joint space preserved), fair (joint space partially narrowed), and poor (loss of joint space). Hips with excellent or good joint congruency were considered an acceptable outcome.

### Surgical technique

#### Incision and approach

In the supine position, the anterolateral S–P incision approach of the hip joint is taken. The skin and subcutaneous are cut in sequence. The lateral femoral cutaneous nerve is exposed and protected along the way. Along the sartorius, rectus femoris, and tensor fascia lata muscles, the surgeon strips the iliac muscle of the medial iliac bone to the quadrilateral and then performs an osteotomy of approximately 2 × 1 cm in the anterior superior iliac spine. Finally, the surrounding muscles were gradually stripped to reveal the ischial notch, ischial spine and ischial minor notch.

#### Osteotomy

Under c-arm fluoroscopy, the surgeon uses a crescent knife to perform incomplete osteotomy (about 1/2 to 1/3) of the proximal ischial branch close to the acetabular groove. After that, the surgeon performed a complete osteotomy of the proximal medial tuberosity of the pubis, then peeled off part of the periosteum of the outer plate of the iliac bone, and performed a vertical osteotomy at the anterior inferior iliac spine to the arcuate line about 1.5 cm. Finally, the osteotomy of the medial quadrilateral, ischial spine, and ischial branch was exposed, and a quadrilateral osteotomy was performed at a distance of about 1.5 cn behind the ischial notch.

### Fix the osteotomy site and close the incision

The surgeon uses 3 bone screws to fix the anterior inferior iliac spine osteotomy site and 2 fix the anterior superior iliac spine osteotomy block. After the surgeon determined that the passive movement of the hip joint was good, the osteotomy angle and the internal fixation under the c-arm fluoroscopy were satisfactory, and then, the incision was closed layer by layer.

### Postoperative care and outcome evaluation

Postoperatively, the surgical hip was kept abducted in a neutral position for two weeks. The patients were encouraged to actively exercise the limb and were instructed to undergo quadriceps femoris and gastrocnemius muscle isometric contraction training and ankle pump exercises. Unilateral DDH patients could get out of bed early to practice walking with two crutches, while bilateral DDH patients were told to walk with help for half a year after the surgery. The patient can bear partial weight 3 months after the operation, and half a year after the operation, consider whether to bear the weight completely according to the healing of the fracture.

Postoperative follow-up was carried out at three days, one week, three months, six months and 12 months. At each appointment, anteroposterior and lateral X-rays were taken for radiographic evaluation. Clinical outcomes were assessed by comparing the HHS and iHOT-12 before surgery and at the latest follow-up appointment.

### Statistical analysis

Continuous variables are expressed as the means and standard deviations, and categorical variables are expressed as numbers and percentages. When preoperative and postoperative radiographic parameters and patient-reported outcomes were analyzed, a t-test was used when they conformed to a normal distribution; the Wilcoxon signed-rank test was used when they did not conform to a normal distribution. Variance inflation factor (VIF) was used to determine whether there is multicollinearity between various indicators. Multivariate logistic regression analysis was used to conduct statistical analysis on the included parameters to predict the risk factors influencing the outcome, and receiver operating characteristic (ROC) curve analysis was performed on the risk factors to determine their sensitivity, specificity and cutoff value. *p* < 0.05 was considered statistically significant, and all data were statistically analyzed with SPSS Software version 23.0 (IBM).

## Results

A total of 59 patients (17 male and 42 female) were included in the analysis. The mean follow-up time was 3.01 ± 1.19 years (1–6 years). The mean age and BMI were 33.61 ± 9.31 years old (range, from 18 to 54 years old) and 22.37 ± 1.84, respectively (Table 1).

**Table 1:** Patient characteristics

Characteristic	
Number of patients (hips)	59 (66)
Age^†^ (year)	33.61 ± 9.31(18–54)
Females (no. [%])	42(71.2%)
Time to latest follow-up (yr)^†^	3.01 ± 1.19(1–6)
Height^‡^ (m)	1.60 ± 0.07
Weight^‡^ (kg)	57.79 ± 7.87
Body Mass Index, BMI^‡^ (kg/m^2^)	22.37 ± 1.84
HHS	
Preoperative^‡^	61.00 ± 16.16
Postoperative^‡^	80.65 ± 7.14
*P* value	< 0.001
iHOT-12	
Preoperative^‡^	60.94 ± 22.41
Postoperative^‡^	87.11 ± 19.98
P value	< 0.001

^†^The values are given as the mean and the standard deviation, with the range in parentheses

^‡^The values are given as the mean and the standard deviation

### Patient-reported outcomes

The changes in patient-reported outcomes were significantly different between the final follow-up and the preoperative results. The patients reported a mean improvement from a 61.00 ± 16.16 preoperatively to a 80.65 ± 7.14 at the last follow-up (*p* < 0.001) on the HHS scale and from 60.94 ± 22.41 to 87.11 ± 19.98 (*p* < 0.001) on the iHOT-12 scale (Table 1). In addition, the comparison between the HSS of the preserved hip group (86.02 ± 3.29) and the symptomatic hip group (74.06 ± 5.99) was statistically significant (*p* < 0.001). The comparison between the iHOT-12 of the preserved hip group and the symptomatic hip group was also statistically significant (*p* < 0.001) (Table 2). By comparing the follow-up times of the preserved hip group and those of the symptomatic hip group, we found that the difference was not statistically significant (Table 2), which indicated that the difference in the follow-up time between the two groups did not lead to bias in the HHS-based results. However, the follow-up times of iHOT-12 got adverse result.

**Table 2:** Patient-reported outcomes at latest follow-up

	Hips. n	Mean postoperative HHS (SD)	*P* value^†^	Time to the latest follow-up of HHS (SD)*	*P* value^†^	Hips. n	Mean postoperative iHOT-12 (SD)	*P* value^†^	Time to the latest follow-up of iHOT-12 (SD)*	*P* value^†^
Hips group			< 0.001		0.181			< 0.001		0.016
Preserved group	40	85.33 ± 3.79		3.13(1.21)		46	95.80(13.88)		3.20(1.25)	
Symptomatic group	26	73.46 ± 4.63		2.71(1.28)		20	67.10(17.48)		2.42(1.08)	

*Time of patient-reported outcomes obtained at the latest follow-up

^†^Independent samples t-tests

### Radiographic parameters outcomes

**Fig. 2:**
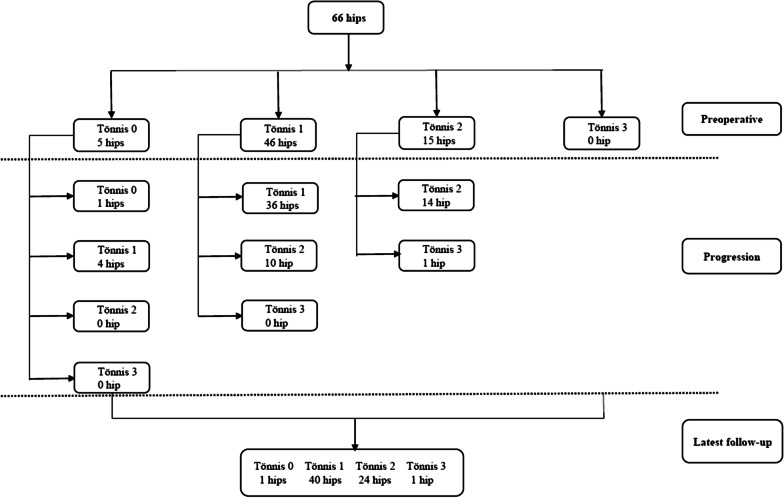
The progression of osteoarthritis according to the Tönnis classification

The progression of 66 hips in terms of Tönnis grade is shown in Fig. 2. After PAO, the hips were classified according to the changes of HHS, and a total of 40 preserved hips and 26 symptomatic hips were documented. 46 preserved hips and 20 symptomatic hips were analysed in the iHOT12-based results. Whether it was evaluated by HHS or iHOT-12, the statistical results showed that postoperative LCEA, Tönnis angle and preoperative poor or fair joint congruency were all statistically significant (Table 3).

**Fig. 3:**
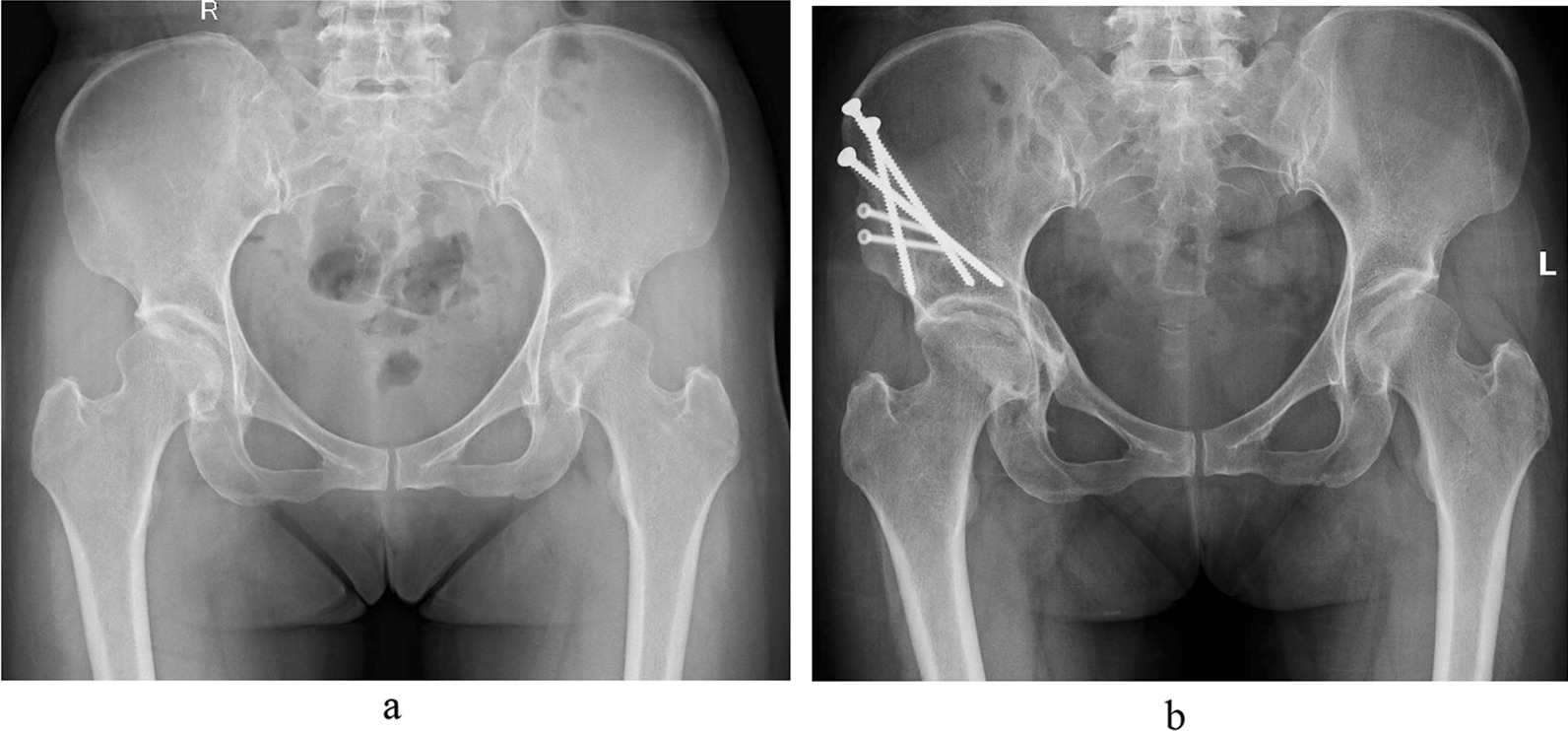
Female, 46 years. **a** Preoperative X-ray. **b** Postoperative X-ray. The preoperative LCEA was 24°, and the postoperative LCEA was adjusted to 55°. The preoperative HHS (iHOT-12) was 67 (58), and the postoperative HHS was 73 (70). The MCID of HHS (iHOT-12) was 6 (12)

**Table 3:** Patient characteristics of cohort

Radiographic parameters	No. (HHS)		*P* Value^†^	No. (iHOT12)		*P* Value^†^
	Preserved (n = 40 hips)	Symptomatic (n = 26 hips)		Preserved (n = 46 hips)	Symptomatic (n = 20 hips)	
Preoperative radiographic parameters						
Acetabular coverage ratio < 75%	40 (100%)	23 (88%)	0.111	46 (100%)	17(85%)	0.025
Sharp angle > 40°	39 (98%)	25 (96%)	1.000	44 (96%)	20 (100%)	1.000
LCEA			0.108			0.128
< 5°	15 (38%)	5 (19%)		16 (35%)	4 (20%)	
5°–20°	21 (53%)	14 (54%)		25 (54%)	10 (50%)	
> 20°	4 (10%)	7 (27%)		5 (11%)	6 (30%)	
Tönnis angle > 10°	39 (98%)	25 (96%)	1.000	45 (98%)	19 (95%)	0.517
Joint congruency poor or fair	11 (28%)	18 (69%)	0.001	13 (28%)	16 (80%)	0.000
Postoperative radiographic parameters						
Acetabular coverage ratio > 75%	40 (100%)	26 (100%)	1.000	46 (100%)	20 (100%)	1.000
Sharp angle < 40°	36 (90%)	25 (96%)	0.655	42 (91%)	19 (95%)	0.988
LCEA			0.000			0.001
< 20°	0	1 (4%)		1 (2%)	0	
20°–38°	27 (68%)	5 (19%)		29 (63%)	3 (15%)	
> 38°	13 (33%)	20 (77%)		16 (35%)	17 (85%)	
Tönnis angle			0.012			0.011
0°–10°	25 (63%)	13 (50%)		30 (65%)	8 (40%)	
− 10°–0°	14 (35%)	6 (23%)		14 (30%)	6 (30%)	
< − 10°	1 (3%)	7 (27%)		2 (4%)	6 (30%)	
Joint congruency poor or fair	7 (18%)	8 (31%)	0.209	9 (20%)	6 (30%)	0.542

^†^Based on univariable comparisons between preserved and symptomatic hips

The radiographic parameters were greatly changed before and after PAO. The acetabular coverage ratio improved from 0.58 ± 0.14 to 0.90 ± 0.09 (*p* < 0.001), the sharp angle improved from 47.78 ± 4.33 to 33.86 ± 4.34 (*p* < 0.001), the LCEA improved from 9.02 ± 13.08 to 38.02 ± 8.28 (*p* < 0.001), and the Tönnis angle improved from 22.82 ± 9.45 to 0.07 ± 7.13 (*p* < 0.001, Table 4).

**Table 4:** Radiographic correction

Characteristic	Mean (SD)*		*P* value^†^
	Preoperative	Postoperative	
Acetabular coverage ratio	0.58 ± 0.14	0.90 ± 0.09	< 0.001
Sharp angle (°)	47.78 ± 4.33	33.86 ± 4.34	< 0.001
LCEA (°)	9.02 ± 13.08	38.02 ± 8.28	< 0.001
Tönnis angle (°)	22.82 ± 9.45	0.07 ± 7.13	< 0.001

*The values are given as the mean and the standard deviation

^†^Based on univariable comparisons between preoperative and postoperative outcome

Risk factors were predicted based on multivariate logistic regression analysis. This study included age, sex, BMI, preoperative and postoperative LCEA, acetabular coverage ratio, sharp angle, preoperative Tönnis grades, Tönnis angle and joint congruency in the risk factor analysis. The results showed that the LCEA (Fig. 3), Tönnis angle and preoperative joint frequency had a significant influence on the outcome, and the remaining factors were not significantly different. The VIFs of LCEA, Tönnis angle and preoperative joint frequency were all 1.79, 1.97 and 1.27 in the HHS-based results and iHOT-12-based results, respectively. The results of The VIFs showed that there was no strong multicollinearity between these indicators. When the postoperative LCEA was > 38°, the risks of an adverse outcome were 16.093-fold higher (odds ratio [OR]: 16.093; 95% CI 1.696–102.788; *p* = 0.003; HHS; Table 5) and 10.854-fold higher (odds ratio [OR]: 10.854; 95% CI 2.520–69.475; *p* = 0.012; iHOT12; Table 5). A Tönnis angle of 10°–0° was a protective factor (odds ratio [OR]: 0.083; 95% CI 0.012–0.554) in the HHS-based results, but not in the iHOT-12-based results. In addition, hips with fair or poor joint congruency were 4.793 times (odds ratio [OR]: 4.793; 95% CI 1.137–20.214; HHS; *p* = 0.004) and 8.960 times (odds ratio [OR]: 8.960; 95% CI 1.892–42.442; iHOT12; *p* = 0.006) more likely to develop negative outcomes.

**Table 5:** The multivariate logistic regression analysis

Variable	HHS^‡^		iHOT12^‡^	
	OR (95% CI)	P value	OR (95% CI)	P value
Postoperative LCEA*		0.013		0.042
< 20°		1.000		1.000
> 38°	16.093(2.520–102.788)	0.003	10.854(1.696–69.475)	0.012
Postoperative Tönnis angle^†^		0.023		0.17
< − 10°		0.925		
− 10°–0°	0.087 (0.012–0.656)	0.018		
Preoperative joint congruency poor or fair	4.793 (1.137–20.214)	0.004	8.960 (1.892–42.442)	0.006

*The statistical results of < 20° and > 38° in postoperative LCEA were compared with 20°–38°

^†^The statistical results of < − 10°and −10°–0° in postoperative LCEA were compared with 0°–10°

^‡^Using HHS and iHOT12 as the outcome indicators, respectively

**Table 6:** Prognostic values of postoperative Tönnis angle, LCEA and

	Cutoff value	AUC (%)	Specificity/sensitivity	Youden’s index
HHS*				
LCEA	38.2	71.1	70%/77%	0.47
Tönnis angle	− 9	62.8	95%/69%	0.64
Joint congruency	–	70.9	72.5%/69.2%	0.417
iHOT12^†^				
LCEA	38.2	75.0	67%/85%	0.52
Tönnis angle	− 9	68.3	94%/65%	0.59
Joint congruency	–	75.9	94%/71.7%	0.657

*ROC analysis with HHS as the outcome indicator

^**†**^ROC analysis with iHOT12 as the outcome indicator

^‡^Joint congruency was divided into two variables, namely joint congruency poor or fair and joint congruency excellent or good. The cutoff cannot be calculated for binary variables

The ROC analysis revealed that the cutoff points for LCEA and Tönnis angle were 38.2 (sensitivity = 77%, specificity = 70%, AUC = 71.1%, log-rank test: *p* < 0.0001, HHS/ sensitivity = 85%, specificity = 67%, AUC = 75.0%, log-rank test: *p* < 0.0001, iHOT-12) and -9 (sensitivity = 69%, specificity = 95%, AUC = 62.8%, log-rank test: *p* < 0.0001, HHS / sensitivity = 65%, specificity = 94%, AUC = 68.3%, log-rank test: *p* < 0.0001, iHOT-12) in the HHS-based results and iHOT-12-based results, respectively. The result showed that preoperative joint congruency (sensitivity = 69.2%, specificity = 72.5%, AUC = 70.9%, log-rank test: *p* < 0.0001, HHS / sensitivity = 71.7%, specificity = 94%, AUC = 75.9%, log-rank test: *p* < 0.0001, iHOT-12) (Table 6).

### Complication

During the follow-up, there were infections in two hips, nonunion of fracture in one hip, minor nerve damage in 10 hips.

## Discussion

DDH has a high incidence in China, with an incidence of 2.9% in Taiwan [30]. If left untreated, joint wear gradually increases and eventually a THA is required [31]. Therefore, hip preservation is a good choice for young patients with DDH. The purpose of hip preservation is to alleviate pain symptoms, slow the progression of osteoarthritis, and delay or even prevent the need for a THA by correcting the hip malformations. Compared with THA, PAO is an ideal choice for young people with symptomatic DDH [32, 33]. Current follow-up results confirmed the efficacy of PAO [5–17]. China was one of the countries to adopt PAO early, but there are few follow-up studies on PAO in the international community. Therefore, we followed patients with DDH after PAO to observe the short-term efficacy of PAO and to predict the factors that affect patient-reported outcomes.

Our study was one of the largest short-term follow-up studies analyzing patient-reported outcomes after PAO performed to alleviate symptomatic DDH. We analyzed the factors that influenced patient-reported outcomes after PAO. In this retrospective study, we found that patients who underwent PAO in our study had good postoperative radiographic parameters and outcome improvement as indicated by patient reports. Additionally, the patients’ progression of Tönnis grade was not obvious. Our data suggest that while hips with fair or poor joint congruency preoperatively and an oversized postoperative LCEA were correlated with unsatisfactory patient-reported outcomes, the proper postoperative Tönnis angle was a positive factor for good patient-reported outcomes in the HHS-based results. When the LCEA and Tönnis angle was 38.2° and -9°, respectively, these are two critical values affected patient-reported outcomes. However, the Tönnis angle was not a related factor for clinical outcome in the Ihot-12-based results. We guess that HHS mainly evaluates joint function, while iHOT-12 mainly evaluates psychological factors, which leads to different results. Based on the results of the nomograms, among hips with poor or fair joint congruency preoperatively treated by surgeons who obtained the improper postoperative LCEAs and Tönnis angles, bad patient-reported outcomes will most likely be obtained.

In a study of 123 hips followed for a mean of 4.3 years after PAO, Trumble et al. [14] reported that 102 hips were preserved, 7 hips required a THA, and 6 subsequent intertrochanteric osteotomies were performed. The patients reported a mean improvement from 65 points preoperatively to 89 points at the latest follow-up, based on HHSs. The latest follow-up radiographic severity of osteoarthrosis, assessed according to Tönnis grade, progressed in only 6 hips. Although this study on PAO revealed good outcomes, only a simple follow-up was performed, and risk factors were not explored in depth. In another prospective, multicenter cohort of 391 hips followed for a mean of 2.6 years after PAO, Clohisy et al. [6] reported that age, sex and BMI were predictive factors of certain outcome measures. This study suggested that the strongest predictors of successful outcomes were female sex, increased age, and a high BMI. In addition, pain, hip function, and quality of life improved after PAO. In our study, 66 hips after PAO performed to alleviate DDH were followed for a mean of 3.01 years. This study showed that sex, age and BMI had no effect on the outcome measures, which may be related to it being a single-center study and including only a limited number of hips.

Wells et al. [16] and Matheney et al. [34] reported that poor or fair preoperative joint congruency is a risk factor for failure, which was consistent with our findings. Another study by Wells et al. [17] analyzed the outcomes of 154 hips followed for an average of 10.3 years and found that excessive postoperative femoral head coverage (LCEA > 38°) was a predictor of failure. Additionally, Albers et al. [5] reported 165 hips after PAO and found that improper acetabular reorientation may accelerate osteoarthritis progression in patients with DDH, and proper acetabular reorientation without introducing femoroacetabular impingement (FAI) improved hip survivorship. To our knowledge, while there have been no other studies to indicate how radiographic parameters can be corrected to obtain favorable outcomes, the current studies showed that an LCEA of > 38° may increase the risk of adverse outcomes. We speculate that the cause of the adverse outcomes may be associated with excessive acetabular coverage, which increases the chance of secondary FAI [5, 17]. Additionally, the correction of the postoperative Tönnis angle after PAO was unclear. According to the experience of the surgeon, a Tönnis angle of 0 ± 10° is in an acceptable range and was achieved in these surgeries. Our multivariate logistic regression analysis found that a Tönnis angle of 10°-0 was a protective factor. Furthermore, considering the results of ROC analysis, our study suggested that a Tönnis angle of -9°-0 may result in better outcomes (Table 5). Not only there is no previous study to support this result, but different surgeons may have different understandings of the correction of the Tönnis angle; therefore, larger multicenter, prospective studies are needed to verify and explain why a postoperative Tönnis angle of -9°-0 is a predictor of successful outcomes.

Our study had some limitations. First, compared with prospective and multicenter studies, the level of evidence in this retrospective and single-center studies is insufficient. All patients underwent surgery by experienced PAO surgeons, which may limit the generalizability of this study. Second, we included 7 patients for analysis who underwent bilateral PAO, which may have affected the results. Third, patients were subjectively selected for inclusion in a retrospective study, which can lead to bias. While we cannot eliminate possible selection bias, strict inclusion criteria can help with drawing significant conclusions. Forth, we only have the parameters of anteroposterior radiographs, but not the relevant parameters of frog-leg and false-profile radiographs. Last, we did not study other factors that influence clinical outcomes, such as Merle d'Aubigné-Postel score, preoperative limp and decreased preoperative internal rotation discovered by Lerch et al. [35] and delayed gadolinium-enhanced MRI of cartilage (dGEMRIC) found by Schmaranzer et al. [36].

Considering these limitations, our study presents novel findings. Short-term follow-up results in the past were mostly reports of patient-reported outcomes, and few studies included baseline or preoperative radiographic parameters in the multinomial logistic regression analysis for the prediction of risk factors. In contrast with these short-term follow-up results, our study not only performed an analysis of the impact of postoperative radiographic parameters on patient-reported outcomes but also performed ROC analysis on the postoperative LCEAs and Tönnis angles to determine their cutoffs.

## Conclusion

Taken together, our results demonstrate marked improvements in patient-reported outcomes after PAO. Among hips with excellent or good joint congruency preoperatively treated by experienced surgeons who obtained the proper postoperative LCEAs and Tönnis angles, good patient-reported outcomes can be expected; the early symptomatic hip rates were low. Continued expansion and follow-up of this study will provide a higher level of clinical evidence to further determine how to improve patient-reported outcomes of PAO. Future studies should pay attention to comparing more postoperative radiographic parameters between asymptomatic and symptomatic hips to determine whether these parameters can be used as factors for predicting failure and as a reference value for PAO.

## Data Availability

The datasets used or analysed during the current study are available from the corresponding author on reasonable request.

## Abbreviations

PAO: Bernese periacetabular osteotomy
DDH: Developmental dysplasia of the hip
HHS: Harris hip score
iHOT-12: International Hip Outcome Instrument-12
ROC: Receiver operating characteristic
THA: Total hip replacement
LCEA: Lateral center-to-edge angle
FAI: Femoroacetabular impingement
dGEMRIC: Delayed gadolinium-enhanced MRI of cartilage
VIF: Variance inflation factor
